# A Case of Posterior Reversible Encephalopathy Syndrome Associated With Pembrolizumab and Cetuximab Combination Therapy

**DOI:** 10.7759/cureus.71867

**Published:** 2024-10-19

**Authors:** Neeja Patel, Rohan Karanth, Dayna J Isaacs, Joss Cohen, Deborah J Wong, Sean D Delshad

**Affiliations:** 1 Internal Medicine, University of California Los Angeles Santa Monica Medical Center, Santa Monica, USA; 2 Neurology, University of California Los Angeles Santa Monica Medical Center, Santa Monica, USA; 3 Hematology and Medical Oncology, University of California Los Angeles Santa Monica Medical Center, Santa Monica, USA

**Keywords:** cancer immunotherapy, cetuximab, hematology-oncology, immune-related adverse event (irae), pembrolizumab side effect, posterior reversible encephalopathy syndrome (pres)

## Abstract

Posterior reversible encephalopathy syndrome (PRES) is a neurologic condition with a constellation of symptoms, including altered mentation, headaches, and often seizures. Immunosuppressive therapies and, more recently, immunotherapy have been identified as risk factors for PRES. We describe the first documented case of PRES associated with a combination of pembrolizumab and cetuximab therapy. This case presentation aims to highlight a rare but potential adverse effect associated with immunotherapy. Awareness of this association and early recognition of symptoms can lead to prompt management and resolution of PRES. Further research is needed to elucidate the mechanisms and risk factors contributing to PRES.

## Introduction

Posterior reversible encephalopathy syndrome (PRES) is a clinical radiographic syndrome characterized by neurologic symptoms and typical findings on neuroimaging. Symptoms include altered mentation, headaches, visual disturbances, and/or seizures [1,2]. Physical exam findings can also include brisk deep tendon reflexes, Babinski signs, and neurologic deficits such as weakness and incoordination of the limbs [3,4]. Neuroimaging most commonly demonstrates symmetric white matter enhancement and/or edema in the posterior cerebrum. The exact pathophysiology of PRES is not entirely understood, but it is believed to involve endothelial dysfunction leading to blood-brain barrier disruption [2,5]. Consequently, uncontrolled hypertension, renal insufficiency, exposure to cytotoxic agents, and immune dysregulation are all risk factors that likely contribute to the development of PRES [6].

Immunotherapy agents such as pembrolizumab and cetuximab are being increasingly utilized in the treatment of various cancers, including squamous cell carcinoma (SCC) of the head and neck [7]. Pembrolizumab is an antibody that targets the immune checkpoint pathway to activate the immune system. The antibody binds to the programmed cell death protein-1 (PD-1) receptor on cytotoxic T cells, blocking their interaction with programmed cell death protein-L1 (PD-L1) on tumor cells, thereby triggering T-cell-mediated anti-tumor responses [8]. Cetuximab is a monoclonal antibody that binds to the epidermal growth factor receptor (EGFR) on tumor cells and blocks the receptor-dependent transduction pathway. This blockade leads to anti-tumor effects such as arresting the cell cycle, inducing apoptosis, suppressing angiogenesis, and preventing metastasis [9]. Anti-EGFR antibodies also induce antibody-dependent cellular cytotoxicity, facilitating immune cell cross-talk, allowing for tumor-antigen-specific cellular immunity and the development of antigen-specific T-lymphocyte responses. The combination of pembrolizumab and cetuximab in patients with SCC of the head and neck has demonstrated a 46% response rate. It is a National Comprehensive Cancer Network guideline-recommended treatment option for patients with SCC of the head and neck [7].

Immunotherapy agents have revolutionized the field of oncology but are also associated with a spectrum of adverse events [10]. The most serious adverse effects of immune checkpoint inhibitor therapy are autoimmune reactions known as immune-related adverse events (irAE), which can affect any organ or system [11]. Furthermore, EGFR inhibitors are commonly associated with cutaneous adverse reactions, headache, diarrhea, electrolyte derangements such as hypokalemia and hypomagnesemia, and infection [12].

Although rare, PRES has been previously associated with both cetuximab and pembrolizumab monotherapy [13-15]. However, to our knowledge, there are no documented cases of PRES associated with combination cetuximab and pembrolizumab therapy. We present the first case of PRES related to combination cetuximab and pembrolizumab therapy.

## Case presentation

A 66-year-old male with advanced SCC of the tongue with metastases to the thoracic spine, right iliac bone, and sacrum, prior pulmonary emboli, and recurrent embolic ischemic strokes presented to the hospital with uncontrolled epistaxis and right arm spasms.

The patient was diagnosed with human papillomavirus (HPV)-related stage III cT4N2M0 poorly differentiated carcinoma of the tongue four years prior. This diagnosis indicated large tumor size, large depth of invasion, and involvement of one cervical lymph node. Notably, the patient was a never-smoker without significant passive exposure, no alcohol use, and was not a candidate for surgery. He underwent definitive chemoradiation, receiving seven cycles of cisplatin therapy with concurrent radiation. Following treatment, a positron emission tomography-computed tomography (CT) scan showed a reduction in the tongue's soft tissue mass and no evidence of fluorodeoxyglucose (FDG)-avid malignancy. Unfortunately, two years after the initial diagnosis, metastases were detected in the T8 vertebra, right iliac bone, and sacrum. A biopsy of T8 confirmed the same malignancy. Subsequently, the patient underwent laminectomy, adjuvant radiation, and eight cycles of carboplatin, paclitaxel, and pembrolizumab therapy, followed by 15 cycles of pembrolizumab maintenance therapy. These cycles were initiated roughly three years prior to this current presentation. Six months later, he developed worsening radiographic bone metastases and possible liver metastases. At that point, the patient was started on combination pembrolizumab and cetuximab therapy, which was two months prior to this current presentation.

Additionally, three months prior to the presented hospital admission, the patient had been transitioned from apixaban to enoxaparin, given the concern for direct oral anticoagulant treatment failure due to recurrent embolic strokes. Extensive evaluations, including transesophageal echocardiogram, cardiac magnetic resonance (MR) imaging, ultrasound duplex of the lower extremities, and telemetry, were negative for other venous thromboembolic disease or embolic sources. However, transcranial Doppler demonstrated ongoing microemboli, so his recurrent embolic strokes were suspected to be due to hypercoagulability of malignancy. Two weeks prior to the current presentation, the patient completed his second cycle of pembrolizumab and cetuximab therapy.

On admission, vital signs were a temperature of 38.6°C, blood pressure of 121/71 mmHg, heart rate of 81 beats per minute, respiratory rate of 16, and oxygen saturation of 97% on room air. The physical examination was unrevealing, with normal bilateral strength, no pronator drift, and an intact mental status. The initial laboratory results on admission are displayed in Table 1.

**Table 1:** Notable initial laboratory results on admission

Pertinent Lab Data	Patient's Lab Values	Reference Range
Hemoglobin	7.7 g/dL	13.5-17.1 g/dL
Anti-Xa level	>2.0 IU/mL	0.6-1.1 IU/mL for twice daily dosing
Magnesium	1.2 mEq/L	1.4-1.9 mEq/L
Corrected ionized calcium	1.07 mmol/L	1.09-1.29 mmol/L
25-hydroxy vitamin D	9 ng/mL	20-50 ng/mL

The patient was started on magnesium and ergocalciferol supplementation to correct electrolyte derangements thought to be causing his muscle spasms. Enoxaparin was held given his epistaxis and supra-therapeutic anti-Xa level. Diagnostics on admission included a CT scan of the brain without contrast, which was negative for acute pathology.

**Figure 1:**
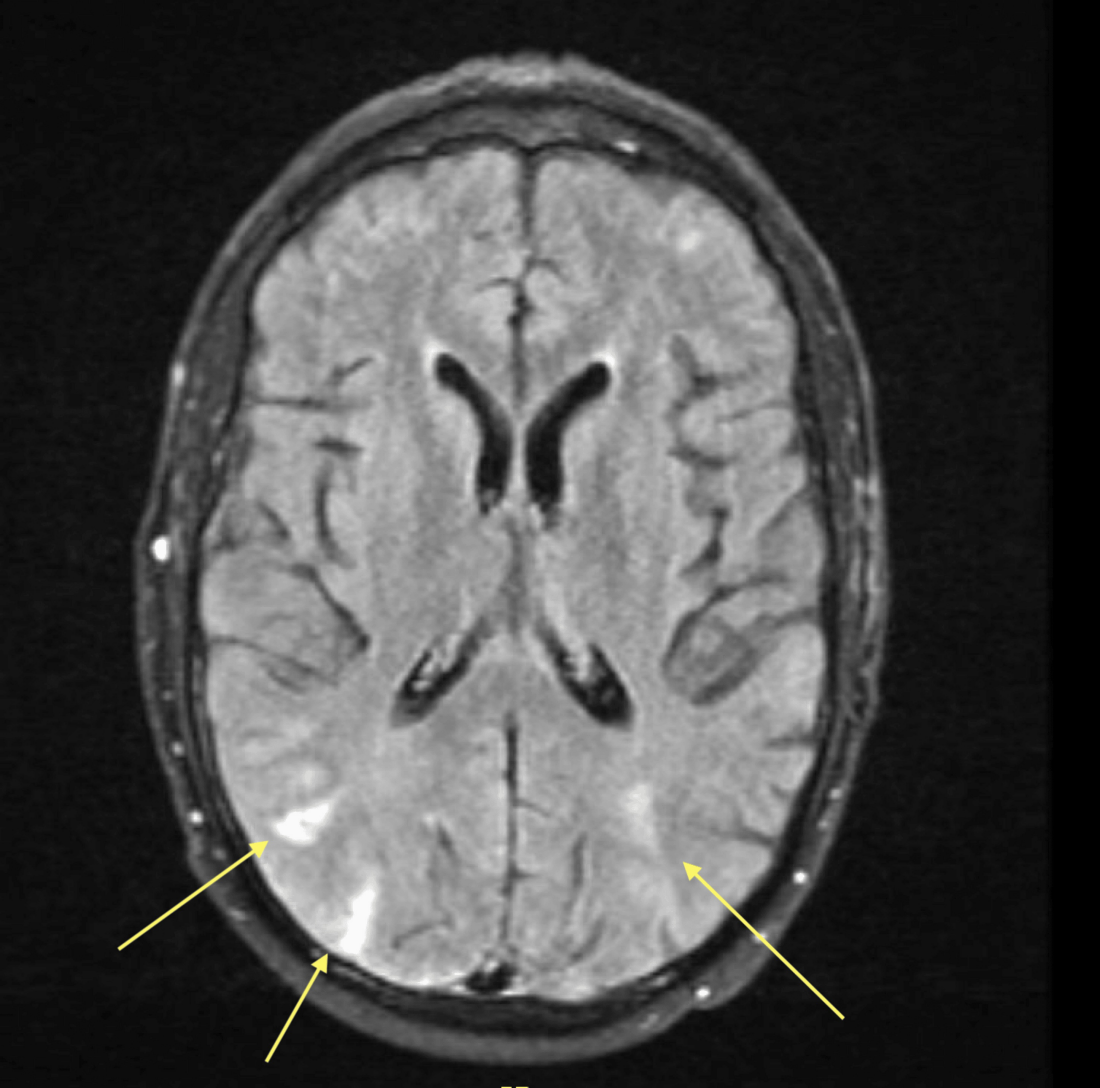
Magnetic resonance imaging of the brain during a code stroke demonstrating bilateral posterior fluid-attenuated inversion recovery (FLAIR) hyperintensities

Within several hours of admission, the patient developed altered mentation and bowel incontinence, prompting the activation of a code stroke. The patient had a National Institute of Health Stroke Scale score of 6 due to aphasia, apraxia, and bilateral arm weakness with pronator drift. MR imaging and angiography of the brain demonstrated new bilateral extensive cortical fluid-attenuated inversion recovery (FLAIR) hyperintensities in the frontal, temporal, parietal, and occipital lobes with questionable foci in the left thalamus and bilateral cerebellum (Figure 1). There was no abnormal restricted diffusion or large vessel occlusion.

**Figure 2:**
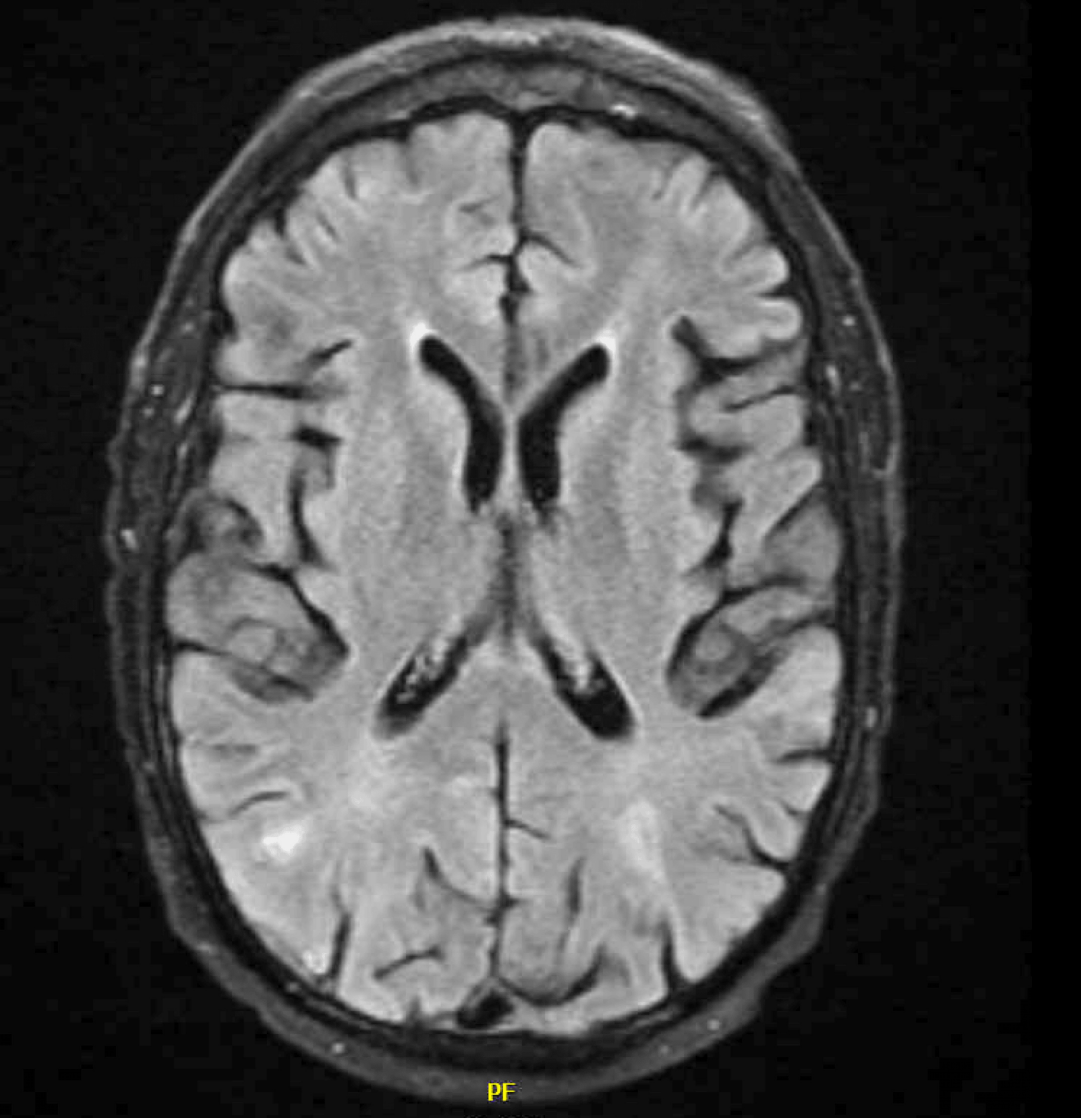
Repeat magnetic resonance imaging of the brain with a resolution of FLAIR hyperintensities FLAIR: fluid-attenuated inversion recovery

Four hours after the code stroke was called, the patient's neurologic deficits resolved, and he returned to his baseline. The patient was diagnosed with PRES based on his neurologic symptoms and posterior hyperintensities seen on imaging. He was treated with supportive measures for his epistaxis and PRES: levetiracetam was initiated for seizure prophylaxis, and continuous electroencephalography (EEG) was negative for seizure activity. Steroid therapy was considered for a potential irAE but ultimately deferred, given his rapid return to baseline. The patient remained normotensive throughout his admission. Subsequent MR brain imaging a week after the initial presentation demonstrated resolution of the posterior hyperintensities that were seen during the acute event (Figure 2).

The patient's epistaxis and muscle spasms improved. However, given his extensive tumor burden, comorbidities, and the PRES diagnosis, causing uncertainty about the safety of continuing immunotherapy, the patient elected to transition to hospice care.

## Discussion

The patient's presentation was consistent with PRES due to acute neurological changes that rapidly resolved, MR imaging showing bilateral posterior FLAIR hyperintensities, and multiple risk factors, including ongoing anti-cancer therapy and multiple cerebral embolic events. Infectious and autoimmune encephalitis were considered as possible etiologies but thought unlikely based on the MRI findings, rapid symptom resolution, and improvement without specific treatment. Immune-mediated encephalitis, a rare but life-threatening complication of immune checkpoint inhibition, was considered but was also unlikely, given the patient's rapid recovery without further immunosuppression, such as steroids.

While PRES typically occurs in the setting of uncontrolled hypertension, increasingly, cases have been attributed to anti-cancer therapies, particularly immunotherapy [16]. In a review of cases of PRES, approximately 75% of patients have moderate to severe hypertension at presentation [17]. However, PRES can occur in normotensive patients, as seen here. Of the cases with normotension, a significant portion occur in patients receiving immunosuppressive or anti-cancer therapies [18].

The patient also reported right upper extremity spasms, which appeared earlier than other neurological symptoms and were thought to be related to low magnesium levels. His hypomagnesemia was likely caused by cetuximab, and magnesium repletion resolved his spasms, making them unlikely to be associated with PRES.

It remains uncertain whether cetuximab, pembrolizumab, or their combination triggered PRES in this patient. Previous literature has documented five cases of PRES associated with pembrolizumab and three with cetuximab [10,13-16,19]. In this case, the patient was receiving both therapies at the time of the PRES episode, so either or both could have contributed. Notably, the patient had tolerated pembrolizumab for over a year without adverse effects.

The exact pathophysiology of PRES remains unknown, but part of the syndrome is believed to involve increased vascular permeability and endothelial dysfunction [20]. Although his anti-cancer therapy may have been the driving factor behind his PRES, his significant hypercoagulability and extensive clot burden may have also played a role in predisposing him to endothelial dysfunction and, subsequently, PRES [2,6]. Given PRES occurred while he was taking both cetuximab and pembrolizumab, both medications were deemed contraindicated moving forward. This case illustrates an important challenge in oncology: rare complications of combination therapies may complicate care, especially when it is unclear which drug is responsible. This can subsequently cause therapeutic uncertainty in determining which oncologic medications are safe for patients to continue for further treatment. Ultimately, it is not entirely clear which drug contributed to this neurologic phenomenon and how much of a role his hypercoagulable state played in causing endothelial dysfunction.

Further research is necessary to clarify the factors predisposing patients to develop PRES, particularly those undergoing combination immunotherapy. Retrospective studies and reviews could help better understand the safety profiles of these regimens. Additional studies might explore the relationship between hypercoagulability, embolic events, and PRES. Due to the rarity of PRES, such investigations are essential to improve our understanding and management of this syndrome.

## Conclusions

We present a novel case of PRES in the setting of a dual immunotherapy regimen: pembrolizumab and cetuximab. Pembrolizumab had been initiated 35 months prior to the patient's admission, with cetuximab initiated two months prior. While PRES is a reported rare adverse effect of both agents as monotherapy, PRES is more commonly reported as an irAE with pembrolizumab.

As combination regimens continue to develop in oncology, clinicians must remain aware of the potential for rare complications. Further studies are recommended to better understand the safety profiles and incidence of adverse events in immunotherapy regimens.
